# Expression of miR-210-3p in the aqueous humor of patients with age-related cataracts and its effect on human lens epithelial cell injury induced by hydrogen peroxide

**DOI:** 10.5935/0004-2749.2022-0274

**Published:** 2023-03-20

**Authors:** Chen Xu, Jianping Xu, Wenlong Zhang, Famang Zheng, Xiangfeng Lou

**Affiliations:** 1 Ophthalmology department, Jinhua Polytechnic, Jinhua, Zhejiang, 321000, China; 2 Cataract specialist, Jinhua Eye Hospital, Jinhua, Zhejiang, 321000, China

**Keywords:** Cataract, Age factors, Aqueous humor, MiR-210-3p, Oxidative stress, Autophagy-related protein 7, Catarata, Fatores etários, Humor aquoso, MiR-210-3p, Estresse oxidativo, Proteína 7 relacionada à autofagia

## Abstract

**Purpose:**

The regulatory effect of microRNA on diseases has been confirmed. This study aimed to evaluate the expression of microRNA-210-3p in age-related cataracts and assess the effect of abnormal miR-210-3p expressions on H_2_O_2_-induced SAR01/04 cells.

**Methods:**

Reverse-transcription quantitative polymerase chain reaction method was performed to assess the levels of miR-210-3p in aqueous humor samples. Receiver operating characteristic analysis was employed to assess the discrimination ability of miR-210-3p between patients with age-related cataracts and healthy people, and Pearson correlation analysis was used to identify the correlation between miR-210-3p and oxidative stress indices such as superoxide dismutase, glutathione peroxidase, malonaldehyde. Cell counting kit-8 assay and Transwell assay were used to estimate the biological function of H_2_O_2_-induced age-related cataract cell model. The levels of oxidative stress indices such as superoxide dismutase, glutathione peroxidase, and malonaldehyde were measured to evaluate the degree of oxidative stress damage in the age-related cataract cell model. The relationship between miR-210-3p and its target gene was verified by luciferase reporter gene analysis.

**Results:**

The miR-210-3p expression was elevated in the aqueous humor of patients with age-related cataracts. A high miR-210-3p expression showed a high diagnostic value for age-related cataracts and was significantly associated with the level of oxidative stress markers in patients with age-related cataracts. The inhibition of miR-210-3p can reverse oxidative stress stimulation and adverse effects on H_2_O_2_-induced cell function.

**Conclusions:**

The results suggested that miR-210-3p could promote cell viability, cell migration, and oxidative stress by targeting autophagy-related gene 7 in in vitro age-related cataract cell model.

## Abstract (pt)

**Objetivo:**

O efeito regulador do microRNA em doenças tem sido confirmado, e este artigo tentou avaliar a expressão do microRNA-210-3p na catarata relacionada à idade e avaliar o efeito da expressão anormal do miR-210-3p em células SAR01/04 induzidas por H_2_O_2_.

**Métodos:**

O método de transcrição reversa seguida de reação em cadeia da polimerase (RT-PCR) quantitativa foi realizado para avaliar os níveis de miR-210-3p em amostras de humor aquoso. Análise de características operacionais do receptor foi feita para avaliar a capacidade de discriminação do miR-210-3p entre pacientes com catarata relacionada à idade e pessoas saudáveis. A análise de correlação de Pearson identificou a correlação do miR-210-3p e índices de estresse oxidativo, como superóxido dismutase, glutationa peroxidase, malonaldeído. O ensaio de contagem de células kit-8 (cck-8) e o ensaio no sistema Transwell foram utilizados para estimar a função biológica do formato de células de catarata relacionada com a idade induzida por H_2_O_2_. Os níveis de índices de estresse oxidativo como superóxido dismutase, glutationa peroxidase e malonaldeído foram detectados para avaliar o grau de dano do estresse oxidativo em formato de células de catarata relacionada à idade. A relação entre miR-210-3p e seu gene alvo foi verificada por análise do gene repórter luciferase.

**Resultados:**

A expressão miR-210-3p foi elevada no humor aquoso de pacientes com catarata relacionada à idade. A expressão miR-210-3p altamente expressiva mostrou alto valor diagnóstico para catarata relacionada à idade e foi significativamente associado ao nível de marcadores de estresse oxidativo em pacientes com catarata relacionada à idade. A inibição de miR-210-3p pode reverter a estimulação do estresse oxidativo e os efeitos adversos da função celular induzida por H_2_O_2_.

**Conclusões:**

Esses dados sugeriram que a expressão miR-210-3p poderia promover a viabilidade celular, migração celular e estresse oxidativo ao direcionar genes ATG 7 relacionados à autofagia em modelo in vitro de células de catarata relacionadas à idade.

## INTRODUCTION

The lens refracts and penetrates the light from outside into the eyes and helps focus the light on the retina^(1)^. However, the lens becomes denser and thicker with age^(2)^. If the lens loses its optical clarity because of some physiological or pathological reasons, a cataract occurs^(3)^. According to the statistics of the World Health Organization, vision loss caused by cataracts accounts for 46% of all cases worldwide^(4)^. Since cataracts are age-related diseases, the burden of blindness increases with population growth and aging. The pathogenesis of cataracts has not yet been fully understood, and studies on the normal physical and chemical properties of the lens and inducements leading to cataracts are ongoing. At present, no effective therapeutic drugs have been established to prevent or delay the occurrence of cataracts, and surgical treatment is still the most effective^(5)^. Therefore, addressing the worldwide problem of cataract pathogenesis is a priority.

MicroRNA (miRNA) is a 22nt length of endogenous RNA^(6)^. It blocks the translation process or directly degrades mRNA by binding to the 3’-UTR of the target mRNA, thus regulating post-transcription and transcription levels, participating in various biological processes, and maintaining a high degree of conservation in the evolutionary process^(7,8,9)^. Recently, increasing studies have confirmed that miRNA participates in the occurrence and development of age-related cataracts (ARCs). For instance, Gao et al. showed that the level of miR-630 in human cataract lens tissues was enhanced compared with that of the normal lens. The reduction of miR-630 levels can inhibit the proliferation and promote apoptosis of human lens epithelial cells^(10)^. Yao et al. reported that low miR-29c-3p levels inhibited cell proliferation and accelerated apoptosis by promoting epithelial-mesenchymal transformation in SRA01/04 cells^(11)^. MiR-210-3p, also known as miR-210, is the most prominent microRNA that is continuously stimulated under hypoxic conditions^(12)^. Thus far, many studies have shown the relationship between miR-210-3p and eye diseases. A study found that miR-210 was augmented in all patients with primary angle-closure glaucoma by sequencing and polymerase chain reaction analysis^(13)^. Yin et al. confirmed the diagnostic significance of highly expressed miR-210 in diabetic retinopathy^(14)^. However, information about miR-210-3p in cataracts is still not fully explored.

In view of the crucial role of miR-210-3p in eye diseases, we hypothesized that miR-210-3p may be involved in the regulation of ARC. Accordingly, this study aimed to evaluate the expression patterns of miR-210-3p in patients with cataracts and explore the pathogenesis of miR-210-3p by constructing an in vitro cataract cell model.

## METHODS

### Participants and samples

This study recruited 70 older patients with non- pathological cataracts (case group). The inclusion criteria were as follows: age ≥55 years, corrected visual acuity of <0.3, and at least one eye has ARC. Another 68 healthy participants matched by sex and age of the case group served as the control group. The exclusion criteria were as follows: (1) patients with glaucoma, macular degeneration, and diabetic retinopathy; (2) patients with a history of cataract surgery; and (3) patients with chronic diseases, such as cardiovascular, liver, and renal diseases. A complete ophthalmic evaluation was performed on all participants, including slit lamp test, ophthalmoscopy, etc. Aqueous humor samples were collected for biochemical analysis, and basic clinical information is summarized in table 1.

**Table 1:** Clinical indicators of the participants

	Participants		
Characteristics	HC (n=68)	ARC (n=70)	p-value
Age (years)	65.53 ± 4.36	64.89 ± 5.12	0.651
BMI (kg/m^2^)	23.77 ± 2.62	23.27 ± 2.81	0.228
Sex (male/female)	38/30	34/36	0.390
SBP (mmHg)	125.83 ± 11.36	126.71 ± 8.79	0.376
DBP (mmHg)	75.35 ± 6.74	75.88 ± 6.42	0.417
TC (mmol/L)	4.88 ± 1.04	4.97 ± 0.79	0.092
TG (mmol/L)	1.31 ± 0.21	1.29 ± 0.33	0.178
FBG (mmol/L)	5.16 ± 0.52	5.11 ± 0.53	0.121
SOD (U/L)	108.07 ± 20.11	98.99 ± 14.20	0.003
GSH-Px (µmol/L)	149.51 ± 19.27	137.35 ± 30.90	0.006
MDA (nmol/L)	3.37 ± 1.07	3.89 ± 0.99	0.004

ARC= age-related cataract; BMI= body mass index; DBP= diastolic blood pressure; FBG= fasting blood glucose; GSH-Px= glutathione peroxidase; HC= healthy control group; MDA= malondialdehyde; SBP= systolic blood pressure; SOD= superoxide dismutase; TC= total cholesterol; TG= triglyceride. Data are presented as mean ± standard deviation (SD).

The aqueous humor collection method was performed as follows: A 1 mL sterile syringe (No. 9 needle) was used to puncture the anterior chamber at a site 1 mm inside the limbus of the cornea, and 0.2 mL of aqueous humor was quickly extracted (2-5 s), without touching the lens, iris, and corneal endothelium. The sample was placed into a silicified Eppendorf tube and stored in a -80ºC refrigerator for later use.

This study was approved by the Ethics Committee of Jinhua Eye Hospital (EC-2020-0118). All research procedures follow the principles of human research according to the Declaration of Helsinki. All the participants provided written informed consent.

### Cell culture and model establishment

Human lens epithelial cells (SRA01/04) were supplied by ATCC (Manassas, VA, USA) and were cultured in Dulbecco’s modified eagle medium (DMEM; Sigma-Aldrich, St Louis, MO, USA) containing 10% fetal bovine serum (FBS; Gibco, Thermo Scientific, Waltham, USA) plus 1% penicillin/streptomycin (Sigma-Aldrich). An in vitro cell model of cataracts was established by H_2_O_2_ induction. According to previous methods, H_2_O_2_ (Sigma- Aldrich) was diluted with DMEM to a final concentration of 200 µM, and cells were then inoculated with this solution for 24 h^(15)^.

### Cell transfection

Cell transfection was conducted to regulate the expression of miR-210-3p in SRA01/04 cells based on a previously published study ^(16)^. The miR-negative control, miR-210-3p mimic, and miR-210-3p inhibitor were purchased from GenePharma (Shanghai, China) and transfected into the SRA01/04 cells at room temperature by Lipofectamine 3000 (Invitrogen, Carlsbad, CA, USA). After cell transfection, the SRA01/04 cells were treated with H_2_O_2_ to induce cell injury.

### RNA extraction and reverse-transcription quantitative polymerase chain reaction (RT-qPCR)

RNAs were isolated by TRIzol reagent (Invitrogen). The isolated RNA was then reverse-transcribed into cDNA using PrimeScriptTM RT Reagent Kit (TakaRa, Dalian, China). Subsequently, the qPCR method was conducted with the SYBR Green I Master Mix Kit (TakaRa) under a 7300 Real-Time PCR System. The 2^-ΔΔC*t*^ method was utilized to detect the gene expression, and U6 was deemed as the internal reference.

### Cell viability assay

Cell Counting Kit-8 (CCK-8) assay was utilized to estimate the SRA01/04 cell viability^(17)^. The CCK-8 working solution (Dojindo, Kumamoto, Japan) was added to the cell culture plate after the cells were treated based on the experimental procedure. Following 2-3 h of incubation away from light, the OD value at 450 nm was detected by a microplate reader (BioTek, Winooski, VT, USA).

### Cell migration assay

Transwell chambers were used to determine cell migration^(18)^. Briefly, cells were collected after being trea ted according to the experimental procedure. The cells were resuspended in FBS-free medium and then inoculated into the upper chamber, and the complete medium was added to the lower chamber. The above transwell chambers were incubated for 48 h and then treated as follows: the migrated cells in the lower chamber were fixed with 4% paraformaldehyde for 15 min and then stained with 0.1% crystal violet for 10 min. Finally, cells were washed with phosphate-buffered saline, and to analyze cell migration, random fields were collected with an inverted microscope.

### Oxidative stress level detection

The activity of superoxide dismutase (SOD) was evaluated by the xanthine oxidase method^(19)^. Briefly, after the cells were completely lysed, the cell supernatant was incubated with the working solution at 37ºC for 30 min. The absorbance at 550 nm was then examined, and the detection result was represented as U/g protein.

The activity of glutathione peroxidase (GSH-Px) was tested by the GSH-Px Detection Kit (Jiancheng Bio, Nanjing, China) according to literature instructions^(20)^. Total protein concentrations in the cell supernatants were measured by a BCA protein assay kit (Beyotime Institute of Biotechnology, China). All steps were conducted according to product instructions, and test results were expressed as µM·mg^-1^ protein.

The malondialdehyde (MDA) levels were examined by enzyme-linked immunosorbent assay according to a published research protocol^(21)^. After complete cell lysis, the supernatant was mixed with the working solution of the kit and heated for 45 min. Subsequently, the sample was centrifuged, and the absorbance value of the product was measured at 532 nm by a spectrophotometer. The concentration of MDA was calculated by the standard curve method and expressed as U/g protein.

### Luciferase reporter gene assay

The binding sites of miR-210-3p and ATG7 were shown by TargetScan 7.0. Fragments of ATG7 3’-UTR with widetype (WT) or mutant type (MUT) miR-210-3p sequence were formed and inserted into pmirGLO vectors to establish ATG7-3’-UTR-WT and ATG7-3’-UTR-MUT. The vectors and miR-210-3p mimic or inhibitor were co- transfected into target cells via Lipofectamine 3000. After 48 h of cell transfection, a dual luciferase reporter assay system was implemented to test the luciferase activity of each group.

### Statistical analysis

Data were analyzed by SPSS software version 18. Data are represented as mean ± standard deviation (SD). Comparisons of different groups were assessed by the Student t-test and one-way analysis of variance. Pearson’s correlation coefficient method was used to detect the correlation between miR-210-3p and oxidative stress indices (SOD, GSH-Px, and MDA). Receiver operating characteristics analysis was performed to estimate the diagnostic value of miR-210-3p for ARC. P<0.05 was considered significant. Each experiment was repeated in triplicate.

## RESULTS

### Baseline characteristics

Clinical information of the ARC group and the control group is shown in Table 1. As shown in the data, significant differences in SOD, GSH-Px, and MDA were found between the two groups (p<0.01). No differences in age, sex, body mass index, systolic blood pressure, diastolic blood pressure, total cholesterol, triglyceride, and fasting blood glucose between the two groups (p>0.05).

### Expression level of miR-210-3p in patients with ARC and its diagnostic value for ARC

**Figure 1:**
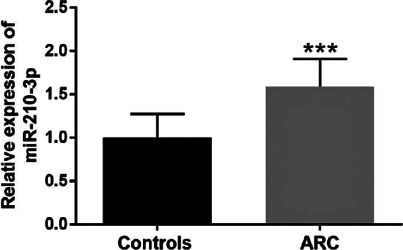
Upregulated miR-210-3p expressions were observed in patients with age-related cataract compared with healthy controls as exhibited by the reverse-transcription quantitative polymerase chain reaction analysis.

**Figure 2:**
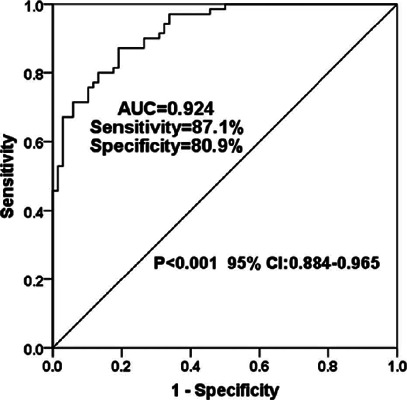
Receiver operating characteristics curve for aqueous humor miR-210-3p as a diagnostic biomarker for age-related cataract.

RT-qPCR analysis was conducted to detect miR-210-3p expression levels in the aqueous humor of all participants. The miR-210-3p level in the ARC group was enhanced compared with that in the control group (Figure 1, p<0.001), which reflected that an abnormal miR-210-3p level might be associated with ARC. As shown in figure 2, ROC analysis revealed an area under the curve of 0.924 at the cutoff value of 1.255, with a sensitivity of 87.1% and a specificity of 80.9%, suggesting the high diagnostic accuracy of miR-210-3p in ARC.

***p<0.001.

### Pearson correlation analysis

As shown in table 2, the analysis of the miR-210-3p level and oxidative stress indices by the Pearson correlation coefficient method revealed that miR-210-3p negatively correlated with the levels of SOD (r=-0.727, p<0.01) and GSH-Px (r=-0.613, p<0.01) but positively correlated with the level of MDA (r=0.678, p<0.01).

**Table 2:** Correlation between miR-210-3p and clinical characteristics

Characteristics	Correlation with miR-210-3p (r)
SOD	-0.727*
GSH-Px	-0.613*
MDA	0.678*

GSH-Px= glutathione peroxidase; MDA= malondialdehyde; SOD= superoxide dismutase.

* Significantly correlated at the 0.01 level (two-sided).

### Effects of downregulated miR-210-3p on sRA01/04 cell function and oxidative stress level

**Figure 3:**
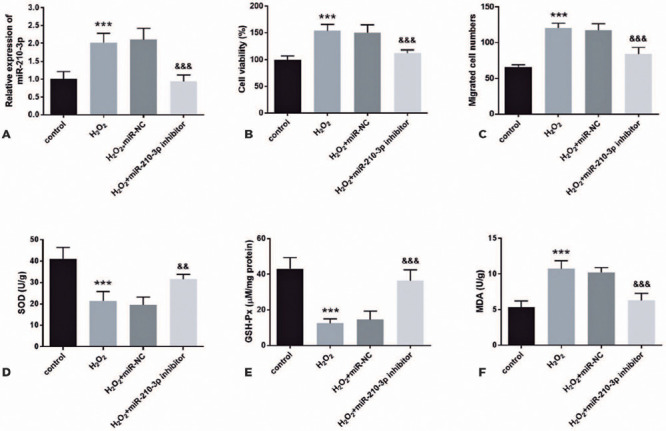
Role of miR-210-3p in H_2_O_2_-induced SRA01/04 cells. The level of miR-210-3p in SRA01/04 cells was significantly downregulated after transfection with the miR-210-3p inhibitor (A). Transfection with the miR-210-3p inhibitor suppressed cell viability (B) and cell migration (C). Transfection with the miR-210-3p inhibitor accelerated the activities of SOD (D) and GSH-Px (E). Transfection with the miR-210-3p inhibitor decreased the level of MDA (F).

To explore the influence of miR-210-3p level on ARC, an ARC cell model was constructed, and miR-210-3p expression was successfully regulated through cell transfection technology. In this study, the level of miR-210-3p increased after H_2_O_2_ induction, and this result can be successfully overturned by the addition of miR-210-3p inhibitor (Figure 3A, p<0.001). The effect of H_2_O_2_ induction on cell biological function was mainly manifested in the promotion of cell viability and migration ability. Interestingly, these adverse effects on cell function were counterbalanced by the transfection of miR-210-3p inhibitor, manifested in the downregulation of cell viability and cell migration (p<0.001; Figure 3B, C). Furthermore, in SRA01/04 cells, the activities of SOD and GSH-Px decreased, and the production of MDA increased following H_2_O_2_ induction. However, the transfection of miR-210-3p significantly protected cells against H_2_O_2_-induced oxidative stress (p<0.001; Figure 3D-F).

***p<0.001, ^&&&^p<0.001, ^&&^p<0.01.

### Validation of the target gene of miR-210-3p

**Figure 4:**
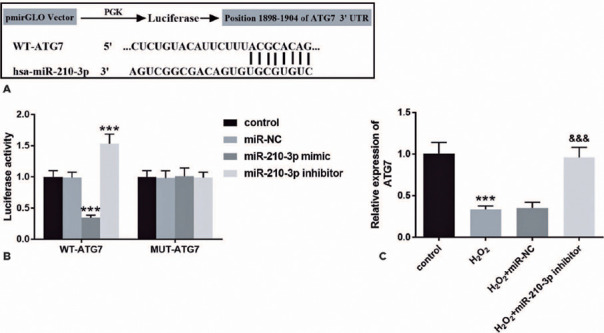
ATG7 is a target gene of miR-210-3p in H_2_O_2_-induced SRA01/04 cells. Complementary sequences of miR-210-3p and ATG7 (A). The interaction between miR-210-3p and ATG7 was detected by the luciferase reporter gene assay (B). Intracellular level of ATG7 in H_2_O_2_-induced SRA01/04 cells (C).

The bioinformatics database TargetScan predicted that miR-210-3p might interact with *ATG7*, and its complementary binding sites are shown in Figure 4A. To verify this hypothesis, the correlation between miR-210-3p and ATG7 was evaluated through the luciferase reporter gene assay. In SRA01/04 cells, cells transfected with miR-210-3p mimic or inhibitor can correspondingly suppress or improve the activity of luciferase in the WT-ATG7 group. However, no effect was found in the MUT-ATG7 group (Figure 4B, p<0.001). Figure 4C revealed that the *ATG7* level declined in H_2_O_2_ -induced SRA 01/04 cells, whereas the expression of *ATG7* was markedly enhanced after the transfection of miR-210-3p inhibitors (p<0.001).

***p<0.001, ^&&&^p<0.001.

## DISCUSSION

Cataracts are a primary cause of blindness globally^(22)^. Studies have shown that ARC is the result of the combined action of multiple factors, and no exact mechanisms are known. With the development of epigenetics in recent years, ophthalmology has begun to study ARC from the perspective of epigenetics, which is expected to put forward a novel method for the clinical treatment of cataracts^(32)^.

This study investigated the biological mechanism of miR-210-3p throughout ARCs. Overall, our data revealed that miR-210-3p was abnormally elevated in the aqueous humor of patients with ARC. Moreover, an in vitro cell study found that the expression of miR-210-3p was significantly augmented in H_2_O_2_-induced SRA01/04 cells. In addition, miR-210-3p overexpression can restrain cell viability and migration, accelerate cell apoptosis, and facilitate H_2_O_2_-induced oxidative stress by regulating *ATG7* expressions in a targeted way.

The disturbance of miR-210-3p has been verified in many diseases. Previous studies have shown that miR-210-3p is involved in atherosclerosis, and its level rises sharply in the first 12 h after macrophages are induced by a high dose of oxidized low-density lipoprotein^(24)^. Moreover, miR-210-3p was elevated in preeclampsia cases^(25)^. In the present study, expressions of miR-210-3p in the serum of the ARC group were upregulated compared with those of the control group. In addition, miR-210-3p was enhanced in H_2_O_2_-induced SRA01/04 cells. These results are consistent with the above conclusions^(13,14)^, revealing that miR-210-3p upregulation may negatively affect the occurrence and development of ARCs.

The pathogenesis of cataracts is influenced by many factors, the most famous of which is oxidative stress. Several studies have shown that oxidative stress leads to lens aging and cataract formation^(26,27)^. Oxidative stress is substantially an imbalance between the production of active substances and the ability of the organism’s protective mechanism to deal with the active compounds and prevent side effects^(28)^. In normal tissues, the production and elimination of reactive oxygen species (ROS) are in a state of dynamic balance. In tissues, ROS are mainly degraded and eliminated by enzymes such as SOD and GSH-Px, and the activity of these enzymes directly reflects the degree of oxidative stress^(29)^. In addition, MDA is the product of lipid peroxidation, and its content can indirectly reflect the degree of oxidative damage of tissues or cells^(30)^. Oxidative stress has been widely considered a major initiator of ARC^(31)^. SOD and GSH-Px are known enzymes with strong antioxidant, anti-aging, and protective effects on cataracts^(32)^. In this study, H_2_O_2_ was used to induce SRA01/04 to construct an in vitro ARC cell model. The results showed that after H_2_O_2_ induction, the cells expressed the characteristics of oxidative stress injury, which demonstrated decreased activities of SOD and GSH-Px and increased production of MDA. However, the inhibition of miR-210-3p not only improved H_2_O_2_-induced oxidative stress but also enhanced cell viability and migration ability and reduced the degree of cell apoptosis. These results directly indicate that inhibiting miR-210-3p expression can significantly impede direct H_2_O_2_-induced cell damage.

The autophagy-related gene (ATG) family has more than 35 members, which play various roles in human diseases^(33)^. Studies have shown that *ATG7* loss can induce oxidative stress and endoplasmic reticulum stress^(34)^. Kozhevnikova et al. found that levels of *ATG7* protein were significantly reduced in rats with advanced retinopathy compared with age-matched control rats^(35)^. Previously, Wang et al. claimed that circ_0004058 inhibited SRA01/04 cell apoptosis by regulating the miR-186/ATG7 axis^(36)^. This study showed that miR-210-3p targeted *ATG7*, and in H_2_O_2_-induced SRA01/04 cells, the *ATG7* level is opposite to that of miR-210-3p. Therefore, the expression of *ATG7* is likely to be negatively correlated with miR-210-3p. In this study, miR-210-3p’s involvement in H_2_O_2_-induced cell function changes and oxidative stress may be achieved by targeting *ATG7*. Obviously, the effect of *ATG7* on cell function and oxidative stress should be verified in H_2_O_2_-induced SAR01/04 cells. Although we have confirmed the abnormal expression and possible pathogenesis of miR-210-3p in the ARC group, this study failed to examine whether the miR-210-3p level in the aqueous humor is consistent with that in the blood, which may be a limitation of this study. Levels of miRNA in the blood may be different from those in other tissues. To test our hypothesis, further investigation of the expression and role of miR-210-3p in the blood and eye tissues, such as lenses, is necessary. In addition, in this study, the results of the clinical study were consistent with those of the in vitro study, which indicated that the designed cell model was reasonable. However, based on the current experimental data, we cannot determine whether the levels of miR-210-3p in other cell types are consistent with those in the aqueous humor. Therefore, incorporating other disease-related cell models is warranted to validate the findings of clinical studies.

In summary, the elevation of miR-210-3p in the aqueous humor of patients with ARC may serve as a candidate diagnostic biomarker to distinguish between patients with ARC and healthy individuals. In addition, in vitro cell experiments indicated that miR-210-3p may accelerate the changes in cell function and oxidative stress injury by regulating *ATG7*. Therefore, our study may provide a new target for the treatment of ARC.
